# Examining the Disproportionate Burden of Microvascular Disease in Women

**DOI:** 10.1007/s11883-025-01310-1

**Published:** 2025-06-12

**Authors:** Meaghan O’Hara, Rukmini Roy, Marie Altenburg, Jeremy Slivnick, Hena Patel

**Affiliations:** Department of Medicine, Unviersity of Chicago, Chicago, IL USA

**Keywords:** Microvascular dysfunction, Coronary artery disease, Cerebrovascular disease, Pulmonary arterial hypertension, Renovascular disease, Preeclampsia

## Abstract

**Purpose of Review:**

Microvascular dysfunction (MD) is a systemic condition implicated in a wide range of pathologies, including ischemic heart disease (IHD), stroke, chronic kidney disease (CKD), dementia, pulmonary arterial hypertension (PAH), and pregnancy complications. MD encompasses conditions characterized by small-vessel obstruction, impaired oxygen delivery, defective clearance of cellular waste, and inadequate gas exchange, ultimately leading to tissue damage and organ dysfunction. This review identifies the role of MD in the pathogenesis of a variety of diseases and across organ systems. It highlights the disproportionate burden of MD in women, with a focus on sex-specific risk factors, especially pregnancy.

**Recent Findings:**

In recent years, there has been increased recognition of the role of MD in the pathogenesis of both cardiac and non-cardiac diseases. Advances in imaging modalities, such as coronary flow reserve assessment and endothelial function testing, have improved the detection of microvascular dysfunction across organ systems. Studies have also highlighted the connection between MD and systemic inflammation, oxidative stress, and hormonal influences, particularly in women. Emerging research suggests that pregnancy-related complications, including preeclampsia and gestational hypertension, may serve as early markers of long-term microvascular dysfunction and cardiovascular disease risk.

**Summary:**

This review focuses on coronary microvascular dysfunction (CMD) in women, with additional discussion of endothelial and microvascular dysfunction in the renovascular, cerebrovascular, and pulmonary arterial systems. This review describes diagnostic and therapeutic approaches for MD in diverse disease contexts and emphasizes the critical need for research to advance diagnostic tools and therapeutic strategies tailored to the unique needs of women.

## Introduction

Microvascular dysfunction (MD) is a systemic disease affecting multiple organ systems, including the cardiovascular, cerebral, renal, reproductive, pulmonary, and ophthalmic systems. MD-associated conditions can cause small-vessel obstruction, impaired oxygen delivery, defective removal of cellular waste products, and insufficient gas exchange, leading to widespread tissue damage and organ failure. Despite overall downtrending deaths from cardiovascular or cerebrovascular disease, deaths remain disproportionately high in women [1]. MD has been proposed as a significant contributor to ischemic heart disease (IHD), obstetric complications, cerebrovascular disease, pulmonary arterial hypertension (PAH), and renovascular disease. In this review, we will unpack the disproportionate burden of microvascular dysfunction in women, and summarize the current understanding of risk factors, pathophysiology, diagnostic challenges, and management of MD across vascular beds.

## Cardiac Microvascular Dysfunction

### Introduction

Heart disease remains the leading cause of mortality among both men and women in the United States [2, 3]. Traditionally IHD has been synonymous with obstructive epicardial coronary atherosclerosis. There is increasing recognition that IHD extends beyond strictly epicardial coronary stenosis, to encompass several heterogeneous conditions, including CMD, that cause insufficient myocardial blood flow (MBF) with or without obstructive narrowing of the epicardial arteries [4, 5].

### Pathophysiology

The coronary microcirculation consists of small blood vessels distal to the epicardial coronary arteries, including the pre-arterioles, intramural arterioles, and capillaries. These vessels account for 90–95% of the total coronary blood flow resistance [6] and are critical for the regulation and maintenance of coronary blood flow. At rest, the arterioles maintain high vascular tone but are capable of significant vasodilation in response to increased myocardial oxygen demand. This vasodilation reduces coronary vascular resistance, enabling healthy coronary circulation to increase blood flow and myocardial oxygen delivery up to five fold above baseline [6, 7].

CMD refers to disorders that impair the functional or structural capacity of the microcirculation, resulting in inadequate coronary blood flow during periods of heightened myocardial metabolic demand and ultimately causing myocardial ischemia [8]. Functionally, CMD can be related to impaired vasodilation and/or excessive vasoconstriction of the microvasculature [8]. Coronary perfusion at the microvascular level is tightly regulated by both endothelium-dependent and endothelium-independent mechanisms, and disruptions in either of these pathways can compromise vasodilation [6, 9]. Structurally, CMD can arise from microvascular architectural changes such as smooth muscle cell hypertrophy and collagen accumulation, with resultant luminal narrowing and impaired coronary blood flow [6, 8]. While both functional and structural pathophysiology of CMD have similar clinical presentations, they have distinct underlying pathophysiologic mechanisms and may therefore require different therapeutic strategies [6]. Systemic inflammation may contribute to the development of CVMD [4, 10, 11, 12, 13]. In one study of patients with CMD but without traditional cardiovascular risk factors, elevated C-reactive protein levels were associated with significantly CFR compared to controls [14].

CMD is thought to be closely linked to three clinical syndromes: angina with nonobstructive coronary arteries (ANOCA), ischemia with nonobstructive coronary arteries (INOCA), and myocardial infarction with nonobstructive coronary arteries (MINOCA) [10]. These conditions describe patients with cardiac chest pain, ischemia, or who have had a myocardial infarction, and yet have no angiographic evidence of significant epicardial coronary stenosis. These clinical syndromes are more prevalent in women than in men, possibly due to the lower prevalence of obstructive epicardial coronary artery disease (CAD) in women [15]. Findings from large cohort studies suggest that women with ANOCA, INOCA, and MINOCA had a high prevalence (40–50%) of abnormal coronary vascular function [16]. This was defined by coronary flow reserve (CFR) and response to acetylcholine or nitroglycerine, which assess endothelial and non-endothelial function in the coronary microcirculation.

CMD is believed to play a critical role in the pathophysiology of two distinct disorders along the spectrum of left ventricular dysfunction: heart failure with preserved ejection fraction (HFpEF) and Takotsubo cardiomyopathy (TTC). Clinical studies and registry data have demonstrated a higher prevalence of HFpEF in women compared to men, with this prevalence increasing with age, reaching 8–10% in women and 4–6% in men aged 80 years or older [17, 18] Although the pathophysiology of HFpEF is not yet fully understood, studies have found that some degree of CMD has been identified in the majority of HFpEF cases: the PROMIS-HFpEF trial, a multicenter prospective observational study, found that 75% of HFpEF patients had CMD, as measured by reduced CFR via Doppler echocardiography [19]. Furthermore, CMD in HFpEF was associated with systemic endothelial dysfunction and elevated levels of NT-proBNP compared to those without CMD [19].

**Fig. 1:**
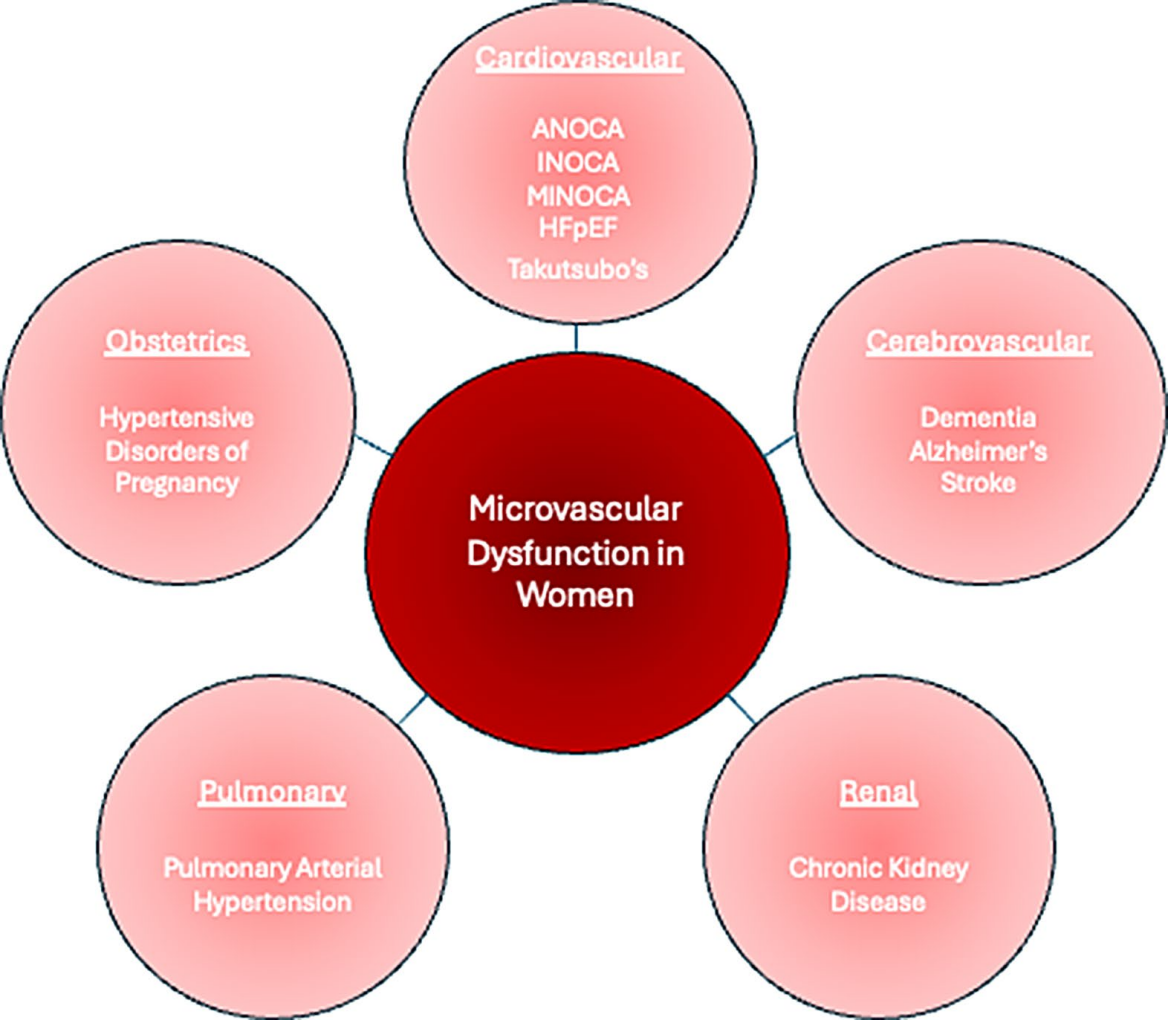
Manifestations of Microvascular Dysfunction in Women

Similarly, CMD appears to play a significant role in the pathogenesis of TTC, a reversible form of cardiomyopathy. The pathophysiology of TTC is complex and not fully understood, though it is generally accepted that many cases are triggered by major emotional or physical stressors. There is a growing body of research which supports a potential association between TTC and CMD. One study assessed coronary microvascular function in TTC patients using invasive coronary angiography and CFR measurements, comparing them to age- and gender-matched patients with INOCA. CMD was present in nearly 80% of patients with TTC, notably more prevalent than in the matched INOCA cohort [20] (See Fig. 1).

### Risk Factors Unique To Women

CMD is more prevalent among women [5, 21, 22]. Men with signs and symptoms of IHD are more likely to undergo coronary angiography, be diagnosed with obstructive CAD, and be treated with percutaneous coronary intervention compared to women [3, 4, 15, 11, 16]. Data from the Women’s Ischemia Syndrome Evaluation (WISE) cohort revealed that up to two-thirds of women referred for angiography due to IHD symptoms did not have significant CAD [11, 23]. Similarly, in a large cohort study of 11,223 patients with stable angina who were referred for coronary angiography, one-third of symptomatic men and two-thirds of symptomatic women were found to have no obstructive CAD [24].

Risk factors for CMD overlap with traditional cardiovascular disease risk factors, but also include aspects specific to female sex, as depicted in Table 1 [12]. Furthermore, there may be sex-specfic factors underlying the pathophysiology of a variety of disease processes which are associated with CMD. Autoimmune and rheumatologic diseases, which disproportionately affect women, are linked to an increased risk of CMD [12]. In one study, 44% of female patients with systemic lupus erythematosus and anginal chest pain exhibited abnormal stress myocardial perfusion on cardiac MRI, despite having no obstructive coronary artery disease [13].

**Table 1:** Female predominant risk factors associated with CMD

Risk Factor	Author, Year
Age	Nichols W.W. et al., 2015
Autoimmune Disease (i.e., psoriasis, systemic lupus erythematosus, and rheumatoid arthritis)	Faccini A. et al., 2016
Smoking	Tran M.V. et al., 2021
Chronic Kidney Disease	Mohandas et al., 2015
Increased level of pro-inflammatory markers	Schroeder J., et al., 2019
Menopause	Stanhewicz A.E., et al., 2018
Migraines	Siak J. et al., 2021
Hypertensive Disorders of Pregnancy	Theberge E.T. et al., 2024
Psychological Stress	Sara J.D.S. et al., 2021

Given the higher prevalence of HFpEF among women and the increased incidence with age, female-specific risk factors and mechanisms have been proposed. In particular, the decline in estrogen levels after menopause is thought to contribute to endothelial dysfunction and CMD, ultimately leading to HFpEF. 17β-estradiol (E2) plays a crucial role in maintaining vascular health by promoting nitric oxide production, enhancing endothelial function, and supporting vasodilation. Postmenopausal women, with reduced E2 levels, experience impaired vascular function, increased vascular remodeling, and a heightened risk of diastolic dysfunction and HFpEF [25]. This hypothesis suggests that estrogen deficiency may contribute to HFpEF pathogenesis, potentially guiding future therapeutic strategies.

Notably, women with HFpEF often experience more severe symptoms, greater congestion, and a lower quality of life compared to men. However, despite these differences, women demonstrate similar rates of HFpEF-related hospitalizations and better survival outcomes than their male counterparts [26].

TTC predominantly affects women, particularly postmenopausal women, with studies reporting that nearly 90% of cases occur in females [27]. This pronounced prevalence among postmenopausal women is thought to result from the loss of estrogen’s protective effects on coronary microcirculation. Similar to mechanisms hypothesized in HFpEF mechanisms, estrogen deficiency following menopause may contribute to the development of CMD, highlighting CMD as a key factor in the pathogenesis of TTC [28].

### Diagnosis

CMD can be diagnosed using both invasive and noninvasive imaging modalities. Invasive diagnostic techniques are currently the gold standard for diagnosis, including the measurement of (CFR) and coronary blood flow (CBF), as well as provocative testing with adenosine and acetylcholine. Provocative testing can elucidate the underlying mechanism of CMD, assessing for endothelium-independent and endothelium-dependent mechanisms during physiological and vasoreactivity testing of CFR and CBF. The endothelial-independent component of the coronary vasculature is interrogated by CFR, as adenosine acts largely independent of endothelium [29]. Acetylcholine testing interrogates the health of the endothelium, with a paracrine effect on the smooth muscle layer in healthy heart [29]. Combined use of vasodilator testing to stratify a nonobstructive CAD offers the optimal accuracy for identifying etiololgies of myocardial ischemia. Studies have shown an adenosine CFR threshold of 2.6 offers excellent specificity with a high positive predictive value ruling in ischemic chest pain while an AChFR of 1.5 has excellent sensitivity with high negative predictive value for ruling this out. Thus, nonobstructive CAD should be investigated by measuring adenosine-mediated vasodilatation and if normal, acetyclcholine-mediated vasodilatation should be performed [29]. In CMD alterations to CFR and CBF are observed in response to these agents, leading to supply-demand mismatch and hypoperfusion in the coronary microvasculature [6, 10]. The safety of provocative testing with acetylcholine to evaluate for vasoreactivity in patients presenting with acute myocardial infarction has been historically questioned. However, a recent prospective study evaluated the safety of provocative testing with acetylcholine in patients presenting with acute myocardial infarction and MINOCA. This study demonstrated that arrhythmic complications (*n* = 2, 5.4%) among patients with positive provocative acetylcholine testing were comparable to patients with spontaneous angina, confirming the safety of provocative testing in patients with acute myocardial infarction [30].

Noninvasive imaging techniques such as positron emission tomography (PET), cardiac magnetic resonance imaging (CMR), and cardiac CT are crucial in diagnosing CMD, quantifying MBF, and assessing the implications for CAD and microvascular angina. PET imaging is the current gold standard to assess MBF and myocardial flow reserve (MFR), using radiotracers like 13 N-ammonia and 82-Rubidium to identify CMD and provide prognostic information [31]. The use of rest and stress PET can quantify myocardial perfusion reserve by using the ratio of MBF during maximal coronary vasodilatation to resting MBF [32]. A myocardial perfusion reserve value of < 1.5 suggests a reduced CFR and MVD. In women without obstructive CAD, PET has shown that this population had a significantly increased adjusted risk of CVD events (*P* < 0.0001, p for interaction = 0.004) [33].

CMR is another promising non-invasive imaging technique in the assessment of CMD, given its high spatial resolution, lack of radiation, and diagnostic accuracy [34]. One study compared the myocardial perfusion reserve index, calculated as the ratio of MBF at hyperemia and rest, between women with CMD and matched controls. Women with CMD had lower myocardial perfusion reserve index values globally in the subendocardial and subepicardial regions [35]. A more recent study, however, found modest inter-scan reproducibility and wide limits of agreement of CMR-derived myocardial perfusion reserve index in patients with suspected CMD. This finding cautions the use of absolute myocardial perfusion index cut-offs in isolation for the diagnosis of CMD, and will require further studies to improve reproducibility [36].

Cardiac CT angiography and CT perfusion imaging are other valuable imaging modalities for perfusion and anatomical evaluation of the coronary vasculature [31]. One prospective trial compared dynamic CT perfusion with rubidium-82 PET in MBF estimation and found CT-derived global MBF was highly correlated with PET MBF (*r* = 0.92, *p* < 0.001).

CMR and cardiac CT offer enhanced spatial and temporal resolution and allow for MBF quantification. However, compared to PET, CMR and cardiac CT show greater variability in measurements due to hardware and methodological complexities [31, 37]. Unlike PET, CMR and CT contrast agents only distribute in plasma rather than the entire vascular space, impacting MBF accuracy [27]. While PET offers robust correction for flow-related inaccuracies, advancements in CMR and CT correction models are still needed. Large-scale randomized trials are essential to establish the clinical utility of these imaging modalities in guiding CMD management and improving cardiovascular outcomes.

There may also be sex-associated differences between CMD imaging modalities. A systematic analysis highlighted significant sex differences in coronary flow parameters, which may affect the comparability of CFR and myocardial perfusion reserve index measurements between men and women [38]. Studies focusing on sex differences in CMD, such as those by Kobayashi et al., show smaller coronary vascular diameters and higher resting coronary blood flow in women [39]. This higher baseline flow can reduce CFR and myocardial perfusion index values in women, as these parameters are calculated using the stress-to-rest perfusion ratio. While some studies report sex differences in resting myocardial blood flow and CFR [40], others find no significant differences [41], emphasizing the need for further research to determine if sex-specific diagnostic thresholds are necessary for noninvasive CMD diagnosis.

*Management*.

Optimal management of CMD remains challenging due to the heterogeneity of underlying mechanisms and the limited number of large, randomized controlled trials targeting this population. One notable study, the CorMicA trial, investigated a stratified medical therapy approach for patients with CMD. This approach was guided by invasive coronary physiology testing, which measured CFR during coronary angiography, followed by vasoreactivity testing using acetylcholine. The trial demonstrated that stratified medical therapy based on coronary function testing significantly improved angina severity and quality of life (QoL) in patients with INOCA when compared to standard care determined by physician preference [42]. However, no differences in major adverse cardiovascular events were observed between the intervention and control groups at six-month follow-up [2, 42, 43]. Improvements in angina and QoL persisted at one year [2, 42, 43]. Based on these results, utilization of coronary physiology testing may be an important step in the diagnosis and management of INOCA.

The ongoing WARRIOR trial is a large, multicenter prospective study aimed at identifying optimal management strategies for patients with undifferentiated ANOCA. This trial compares an intensive medical regimen—including low-dose aspirin, high-intensity statin therapy, and maximally tolerated angiotensin-converting enzyme inhibitors or angiotensin receptor blockers—with standard care. The primary endpoints of this study are all-cause mortality and nonfatal cardiovascular events, and the results may provide further insight into the management of these patients [6].

## Obstetric Microvascular Dysfunction

### Introduction

In addition to the known risk factors discussed earlier in this review, coronary ischemia and endothelial dysfunction appears to be amplified in women with hypertensive disorders of pregnancy and gestational diabetes mellitus in the peripartum and postpartum period [44, 45, 46].

Preeclampsia is a hypertensive disorder of pregnancy affecting 5–8% of pregnancies worldwide [47]. Present data suggests that subclinical dysfunction of the maternal microvascular beds occurs during pregnancy and may precede the development of cardiovascular, renal, hepatic, and other sequelae of MD seen in pregnant women with preeclampsia [48]. In a meta-analysis of nearly 20,000 women from the UK Biobank over 20 years after giving birth, hypertensive disorders of pregnancy were associated with impaired microvascular indices when controlling for blood pressure, arterial stiffness, and cardiometabolic risk factors. This suggests that hypertensive disorders of pregnancies are associated with microvascular aging and may contribute to CVD long after delivery [49]. In another study of 91 individuals who underwent myocardial contrast echocardiography to quantify myocardial perfusion at rest and during dobutamine stress highlighted that individuals with coronary MD had a higher proportion of HDP and higher pre-pregnancy body mass index, baseline heart rate, and hemoglobin A1c compared with those without MD (46.2% versus 16.7%; *P* = 0.026) [50]. It is unclear whether multiple gestational disorders have a cumulative effect, and larger trials are needed, but a small study of 24 patients with combined preeclampsia and GDM had a higher prevalence of CMD (91% vs. 5.6%, *p* < 0.001) than patients with isolated GDM (55%, *p* = 0.01) [46].

### Pathophysiology

Although the exact mechanism of MD in preeclampsia is unknown, a proposed mechanism is that uterine artery remodeling and placental ischemia occurs early in pregnancy, triggering the release of anti-angiogenic factors which enter the maternal circulation and mediate their effects on systemic endothelial and vascular muscle cell function in non-reproductive vascular beds [48]. Myocardial dysfunction has largely been recognized among women who have experienced coronary microvascular dysfunction as mentioned earlier in this review. Outside of the myocardial microvasculature, endothelial dysfunction in preeclampsia also impacts the renal vasculature, causing significant degradation of intraglomerular vascular beds, a process described as endotheliosis, and vasospasm within the renal microvasculature [51]. This results in reduced renal blood flow and a lower glomerular filtration rate in women with preeclampsia compared to women with normal pregnancies [51]. Similarly, the hepatic vasculature is affected in 12% of patients with preeclampsia, often known as HELLP syndrome (Hemolysis, Elevated Liver Transaminases, Low Platelet count), and is associated with an increase in anti-angiogenic, inflammatory, and vasoconstricting factors with subsequent endothelial injury, spasm, and fibrin deposition throughout the hepatic microcirculation [52]. In women with preeclampsia with severe features, microvascular dysfunction also has been shown to impact the cerebrovascular microcirculation, with MRI revealing widespread ischemic injury, micro-hemorrhages, and cerebral edema [48]. GDM might also be associated with MD. In one study, women with GDM had a 70% higher incidence of cardiovascular disease compared to their normal cohorts [53]. In another small study using laser Doppler fluximetry to assess microvascular reactivity, an early marker of endothelial dysfunction, there were no significant differences between patients with GDM and normal controls [54].

### Diagnosis and Management

The diagnosis and management of MD in women with a history of pregnancy complicated by hypertensive disorders or diabetes remains variable. Both invasive and non-invasive diagnostic modalities are infrequently used in pregnancy due to risks. Within these limitations, current data suggests that MD developed during pregnancies affected by hypertensive disorders such as preeclampsia may have adverse outcomes long-term [49]. Management of preeclampsia currently includes aspirin and statin therapy, which are thought to target the endothelial mechanics mediating the maternal MD during preeclampsia. The mechanisms underlying these treatments are still hypothetical and remain unexplored in in vivo human trials [48].

## Cerebrovascular Microvascular Dysfunction

### Introduction

A widespread spectrum of cerebrovascular disorders can be seen in women such as intracranial atherosclerosis, embolism, aneurysms, reversible cerebrovascular vasoconstriction, and cerebral venous sinus thrombosis [55], as well as long-term vascular complications such as dementia [56] or even late-onset depression [57]. The heart and brain have similar vascular structures, and like the coronary microvasculature, the cerebral microvasculature is involved in the regulation of many processes, including cerebral perfusion, neurovascular coupling, blood-brain barrier permeability, and cerebral autoregulation [58]. Smaller studies utilizing SPECT perfusion imaging have shown significant associations between myocardial endothelial dysfunction and abnormal perfusion with abnormal cerebral blood flow [59], strengthening the argument that endothelial dysfunction and its consequences extend beyond coronary ischemia. Disruptions in the endothelial tissue of the microcirculation can have devastating downstream effects and appears to be disproportionately prevalent among women [55].

The age-adjusted stroke death rate in the US increased between 2011 and 2021 by 8.4% (from 37.9 per 100,000 to 41.1 per 100,000), whereas the actual number of stroke deaths increased 26.3% (from 128,932 to 162,890 deaths) [3]. Specifically in women, from 2011 to 2013 to 2020–2022, stroke prevalence increased 9.3% among women compared to 6.2% among men [1]. Additionally, women have a high mortality related to ischemic stroke, and worse QoL with more severe disability after ischemic stroke. They have a longer time from symptom onset to hospital arrival, more delays in thrombolysis, and are less likely to receive appropriate diagnostic evaluation with head imaging (CT or brain MRI) than men [55], although whether these differences are sex-related or age-related is not yet fully known [60].

### Pathophysiology

The pathophysiology behind this sex-specific difference in cerebrovascular disease risk is not fully understood, but MD might play a role. Limited studies have suggested that age-related changes in vascular function include endothelial dysfunction, which progresses at a faster rate in women than men, and may contribute to higher rates of hypertension seen in women than men after the age of 65 [61], contributing to a higher risk of stroke among women compared to men. Another study of 2952 subjects assessed endothelial function using flow-mediated dilation utilizing Doppler flow velocities at baseline and during peak hyperemic flow. They found that lower flow-mediated dilation values were significantly associated with an increased incidence of total stroke, reporting a hazard ratio (HR) of 2.13 (95% CI: 1.48–3.07, *p* < 0.001) [62], indicating that impaired endothelial function may be a strong risk factor for stroke. Women specifically showed a significant association between impaired endothelial function and stroke risk, with a hazard ratio of 0.91 (95% CI: 0.82–0.94, *p* = 0.003) [62]. The peripartum and postpartum periods have also shown higher risk of stroke, specifically in women with gestational diabetes and preeclampsia, which were discussed earlier in this review [55].

### Risk Factors Unique To Women

Traditional risk factors confer a higher risk of stroke in women than in men, including migraine with aura (2.5-fold higher odds of stroke), obesity (61.8% age-adjusted prevalence of abdominal obesity in women with a measurable increase in stroke risk with each unit of increase in waist circumference), metabolic syndrome (trend to higher stroke risk in women), atrial fibrillation (higher stroke risk in women > 75 years than men even after adjustment for comorbidities), and hypertension (higher risk of first stroke with hypertension) [55]. This suggests there may be unique factors related to female sex which impart a higher native risk for cerebrovascular disease beyond traditional risk factors.

### Diagnosis and Management

Great diagnostic challenges remain when evaluating MD in the cerebrovascular system, as traditional imaging examinations such as CT or CTA have a limited ability to evaluate cerebral microvasculature, as they are limited by onset time and lesion size. The gold standard for diagnosing vascular disease includes digital subtraction angiography which can display smaller arteries (DSA), but cannot display blood vessels < 850 μm, and is limited by its invasive nature utilizing ionizing radiation [63]. The most reliable diagnostic method remains magnetic resonance imaging (MRI) utilizing T1, T2, FLAIR, diffusion-weighted imaging, MRI-sensitive weighted imaging, and gradient echo imaging [63]. Studies are underway to identify the early imaging characteristics of cerebral small vessel disease (cSVD) which will improve diagnostic accuracy for cSVD and support longitudinal studies that track changes in radiological markers. At this time, no treatments are available specific to cSVD in women, beyond prevention and treatment of modifiable risk factors.

## Renovascular Microvascular Dysfunction

### Introduction

Similarly to the cerebral and coronary microcirculation, the kidneys are highly vascular organs that receive approximately 20% of the body’s total cardiac output. The microcirculation of the renal system plays a crucial role in sustaining the glomerular filtration rate by modifying renal blood flow and intraglomerular pressure. CKD is associated with MD, which can cause dysregulation of blood flow causing abnormal vasoreactivity, fibrosis, or rarefaction [64]. There is some evidence that pathogenic mechanisms other than obstructive CAD may contribute to excess CV mortality in those with CKD: traditional cardiac risk factors do not account for the majority of associated with CKD, and revascularization or risk reduction have had little success preventing adverse cardiac events particularly among dialysis patients [65].

The WISE cohort demonstrated that CKD is more prevalent among women, who also have a higher mortality related to the disease [66]. While CKD affects 30 million US adults or about 15% of the US adult population, data from the CDC shows 18% of women are diagnosed with CKD compared with 13% seen in men. Current data also illuminates significant differences in sex-related hospitalization rates with an average of 2.08 hospitalizations per year for women versus 1.68 hospitalizations per year for men requiring dialysis, even when age is accounted for [67]. A regression analysis of a multicenter, prospective, cohort study of women with suspected ischemia undergoing coronary angiography, showed a correlation between eGFR and CFR (*r* = 0.22; *P* = 0.002), suggesting a possible element of microvascular dysfunction contributing to the burden of renal insufficiency in women [65].

### Pathophysiology

A current knowledge gap exists regarding the pathophysiology involved in MD of the renal system leading to CKD, specifically among women. A multi-study review of microvascular dysfunction in CKD revealed possible mechanisms including abnormal vasoreactivity, fibrosis driven by vascular-renal signaling, or loss of renal microvasculature associated with kidney disease, though these differences were not sex-specific [64]. While some studies have shown an elevation in biomarkers of inflammation such as IL-6, serum amyloid A, and acute phase reactant C among individuals affected by CKD, it is unclear whether the disproportionate prevalence of CKD among women is related to elevations in particular biomarkers of inflammation [68].

### Risk Factors Unique To Women

Beyond traditional risk factors for CKD such as diabetes mellitus, hypertension, and obesity, there is a paucity of data regarding specific risk factors unique to women in relation to endothelial dysfunction of the renal vasculature. Risk factors unique to women with renal insufficiency include antepartum and peripartum complications, specifically pre-eclampsia, gestational diabetes, and hypertensive disorders of pregnancy as previously discussed in this review [48]. Additional studies are needed to gain further insight on risk factors for MD and renal dysfunction in women.

### Diagnosis and Management

As with coronary and cerebral MD, noninvasive imaging modalities including CT and MRI are the mainstay of the diagnosis of renal microvascular dysfunction [69]. However, they currently lack the resolution for adequately imaging microvasculature in vivo. Even blood oxygen dependent MRI requires established pathological areas to further evaluate, which limits its diagnostic utility. Due to these limitations, renal flow reserve has been explored as an alternate modality, similar to CFR. In a small study of 28 normotensive patients, quantitative angiographic measurements of the renal artery were obtained along with renal artery pressure and flow velocity after injection of various hyperemic agents, with the goal to develop renal flow reserve as an alternate diagnostic modality for renovascular disease [70]. However, unlike coronary flow reserve, renal flow reserve has not yet been standardized or shown to predict adverse cardiovascular or renal outcomes, and remains an area needing further research [70]. At this time there are no sex-specific differences between diagnostic modalities for renovascular disease.

Treatment of microvascular dysfunction in CKD has not yet been clearly defined. Prior studies have focused mainly on treatments for coronary MD seen among women [71], which may have downstream effects on the renal microvasculature due to the role of MD in multiple organ systems. A significant current area of research has involved VEGF, which is a proangiogenic mediator that plays a critical role in the maintenance of peritubular capillaries and promotion of endothelial survival. Limited data utilizing a porcine model of renal artery stenosis has demonstrated preserved renal microvasculature and function in those injected with VEGF [72]. Future studies are needed to help further explore specific diagnostic modalities and management strategies of MD in the renal vasculature.

## Pulmonary Arterial Microvascular Dysfunction

### Introduction

Pulmonary hypertension (PH) encompasses a heterogeneous group of disorders characterized by elevated mean pulmonary artery pressure (mPAP > 20 mmHg) at rest, confirmed via right heart catheterization [73]. Current guidelines classify PH into five distinct groups based on underlying etiology and pathophysiology. Group 1 PH, or pulmonary arterial hypertension (PAH), specifically affects the precapillary pulmonary arterioles, leading to increased pulmonary vascular resistance, vascular remodeling, and elevated pulmonary pressures, eventually resulting in right heart failure and death [74]. Endothelial dysfunction is a key driver of PAH, with additional contributions from smooth muscle proliferation and increased pulmonary arteriole contractility [75–77]. Despite advances in therapy PAH remains a debilitating disease with poor long-term survival: 5-year survival rates range from 21 to 75% in different disease registries [78, 79].

### Risk Factors Unique To Women

PAH is a rare condition, with an estimated prevalence of 10–50 cases per million and an estimated annual incidence of 5–10 cases per million [80, 81]. Multiple registries have shown a higher incidence of PAH in females. The REVEAL registry (*n* = 2525, 2006–2007) reported a 3.8:1 female-to-male ratio, while a French registry (*n* = 674, 2002–2003) noted a 1.9:1 ratio [80, 82]. More recent data from a U.S. registry (*n* = 499, 2015–2018) mirrored REVEAL’s findings with a 4:1 female-to-male ratio [83].

Despite the higher prevalence of PAH in women, females tend to have better outcomes and survival compared to males, a phenomenon referred to as the “estrogen paradox” [84, 85]. This suggests that sex hormones may influence the pathogenesis of PAH differently between the sexes [78]. One factor contributing to improved survival in females is likely better right ventricular function noted in women. Studies have shown that females experience improved right ventricular ejection fraction after treatment, whereas males often exhibit a decline, correlating with poorer survival outcomes [85]. Pregnancy in women with PAH, however, is associated with high maternal and fetal mortality due to the hemodynamic burden imposed on the already compromised RV, making pregnancy management and family planning critical in this population [84, 86].

### Diagnosis and Management

Current therapies for PAH cause pulmonary vasodilation and fall into three main categories: prostacyclin analogues, endothelin receptor antagonists, and a guanylate cyclase stimulator [87]. These therapies cause vasodilation by targeting 3 different pathways that are implicated in PAH. Combination therapy targeting multiple pathways, has shown improved survival over treatment with monotherapy and is thus preferred [87]. It has been reported that females have superior clinical benefit to endothelin receptor antagonists compared to men, as measured by improvement in 6-minute walk distance [88]. Despite its increased prevalence in females, there are currently no treatments targeted specifically towards PAH in women. This could present as a potential future therapeutic strategy.

## Conclusion

Microvascular dysfunction can impact various organ systems, including the cardiovascular, cerebral, pulmonary, reproductive, and renal systems, and has significant clinical consequences. The detection of MD in one organ system may suggest a broader, systemic process involving multiple microvascular beds, such as retinal microvascular dysfunction being linked to both cardiovascular and obstetric microvascular dysfunction [89, 90]. However, there are still considerable gaps in our understanding of MD across different organ systems, including its clinical implications and optimal therapeutic strategies. Further research is needed to elucidate the shared pathophysiology of MD as a systemic disease, as well as the risk factors unique to women, which are just starting to be appreciated in clinical research. Addressing these knowledge gaps may pave the way for the development of targeted, sex-specific therapies for MD.

## Key References

- Smilowitz NR, Toleva O, Chieffo A, Perera D, Berry C. Coronary Microvascular Disease in Contemporary Clinical Practice. Circ Cardiovasc Interv. 2023 Jun;16(6):e012568.
  - Review of the pathophysiology, diagnosis and therapeutic guidelines for coronary microvascular dysfunction.
- Stanhewicz AE, Nuckols VR, Pierce GL. Maternal microvascular dysfunction during preeclamptic pregnancy. Clin Sci. 2021 May 14;135(9):1083–101.
  - Review of maternal microvascular dysfunction in preeclampsia, highlighting its potential contribution to disease pathophysiology.
- 2023 AHA/ACC/ACCP/ASPC/NLA/PCNA Guideline for the Management of Patients With Chronic Coronary Disease: A Report of the American Heart Association/American College of Cardiology Joint Committee on Clinical Practice Guidelines.
  - Current AHA guidelines for management of Coronary diseae.
- Steinberg RS, Dragan A, Mehta PK, Toleva O. Coronary microvascular disease in women: epidemiology, mechanisms, evaluation, and treatment. Can J Physiol Pharmacol. 2024 May 10 https://cdnsciencepub.com/doi/full/10.1139/cjpp-2023-0414.
  - Review of epidemiology, mechanism, evaluation and treatment of coronary microvascular disease in women.

## Data Availability

No datasets were generated or analysed during the current study.

## Abbreviations

ANOCA: Angina with nonobstructive coronary arteries
CAD: Coronary artery disease
CBF: Coronary blood flow
CFR: Coronary flow reserve
CKD: Chronic kidney disease
CMD: Coronary microvascular disease
CMR: Cardiac magnetic resonance imaging
CCT: Cardiac Computed tomography
CTA: CT angiography
HFpEF: Heart failure with preserved ejection fraction
INOCA: Ischemia with nonobstructive coronary arteries
IHD: Ischemic heart disease
MBF: Myocardial blood flow
MD: Microvascular dysfunction
MINOCA: Myocardial infarction with nonobstructive coronary arteries
PAH: Pulmonary arterial hypertension
PET: Positron emission tomography
VEGF: Vascular endothelial growth factor
QoL: Quality of life
WISE: Women’s Ischemia Syndrome Evaluation
